# An Improved Composition of CoFeSiB Alloy for Orthogonal Fluxgates

**DOI:** 10.3390/s22062162

**Published:** 2022-03-10

**Authors:** Mattia Butta, Michal Janošek, Jakub Pařez, Alexander Valeriano Inchausti, Horia Chiriac

**Affiliations:** 1 Faculty of Electrical Engineering, Czech Technical University in Prague, 166 27 Prague, Czech Republic; janosem@fel.cvut.cz (M.J.); parezjak@fel.cvut.cz (J.P.); 2 Institute of Material Science of Madrid—CSIC, 28049 Madrid, Spain; alexvale@ucm.es; 3 National Institute of Research and Development for Technical Physics, 700050 Iasi, Romania; hchiriac@phys-iasi.ro

**Keywords:** fluxgate, magnetic sensors, noise, amorphous magnetic wire, magnetostriction, annealing

## Abstract

Orthogonal fluxgates in fundamental mode based on ${\left({\mathrm{Co}}_{0.94}{\mathrm{Fe}}_{0.06}\right)}_{72.5}{\mathrm{Si}}_{12.5}B_{15}$ cores have recorded very low noise in literature, especially if Joule annealing is performed on the core for a short period of time. However, for annealing time longer than 20–30 min, the noise of the sensor has a tendency to increase. In this work, we investigated this phenomenon, and we have found its origin in a monotonic increase of magnetostriction during the annealing process. We show that the wires with vanishing magnetostriction in their as-cast form exhibit positive magnetostriction after long-time annealing (more than 30 min), which increases the noise of the sensor. After researching the effect of the magnetostriction after annealing on the noise, we propose an alloy with a reduced amount of iron. Less iron leads to a larger as-cast negative magnetostriction, which becomes almost zero after long-time annealing (60 min), bringing further reduction of noise. We prove this effect on two wires from two different manufacturers, although with the same composition. The noise decrease with prolonged annealing is mainly observable in the low-frequency region: at 100 mHz, the noise of a single-wire sensor decreased from $20\;\;\mathrm{pT}/\sqrt{\mathrm{Hz}}$ to $6\;\;\mathrm{pT}/\sqrt{\mathrm{Hz}}$ when the annealing time was prolonged from 10 to 60 min.

## 1. Introduction

Cobalt-rich amorphous magnetic wires have been produced for decades using the rotating-water quenching method [1]. A master alloy with the desired composition is melted in a quartz nozzle by eddy currents in argon atmosphere and solidified by ejecting it on a spinning wheel covered with a water layer. If the cooling process is fast enough, the wire is solidified in its amorphous form. This brings excellent magnetic properties, such as low coercivity (tens of A/m [2,3]) and vanishing magnetostriction ($\lambda_{S}\approx 10^{-7}÷10^{-8}$ ) [4,5]. These characteristics make Cobalt-rich wires ideal for different magnetic sensors [6,7,8,9,10,11,12]. Among them, orthogonal fluxgates in fundamental mode [13] have shown a remarkable low noise due to the mechanism that keeps their magnetization always in a saturated state, reducing the Barkhause noise. For instance, magnetometers based on amorphous CoFeSiB microwire exhibited a noise of only $750\;\;\mathrm{fT}/\sqrt{\mathrm{Hz}}$ at 1Hz [14], which is superior to the best reported noise of any second harmonic fluxgate so far [15,16].

Similar sensors, based on a magnetic wire and a pick-up coil, have been presented under the name of off-diagonal GMI: in this case, the best noise proposed is around $30\;\;\mathrm{pT}/\sqrt{\mathrm{Hz}}$ at 10 Hz in [17], and improvements have been shown in [18] with $2\;\;\mathrm{pT}/\sqrt{\mathrm{Hz}}$ at 10 Hz and above $4\;\;\mathrm{pT}/\sqrt{\mathrm{Hz}}$ at 2 Hz. Another sensor based on the same approach is proposed in [19]. In this case, the composition of the glass coated wire is ${\mathrm{Co}}_{67}{\mathrm{Fe}}_{38.85}{\mathrm{Ni}}_{1.45}B_{11.5}$${\mathrm{Si}}_{14.5}{\mathrm{Mo}}_{1.7}$ and the noise achieved is $100\;\;\mathrm{pT}/\sqrt{\mathrm{Hz}}$ at 1 Hz. A full description of the noise dependence of these sensors on the conditioning parameters is given in [20]. Yet another off-diagonal GMI sensor was presented in 2017 based on CoFeSiB, reaching a noise of $50\;\;\mathrm{pT}/\sqrt{\mathrm{Hz}}$ at 1 Hz [21], later reduced to about $40\;\;\mathrm{pT}/\sqrt{\mathrm{Hz}}$ at 1 Hz [22]. It has been shown that, when based on glass-coated microwires, it is useful to reduce the glass thickness by etching it, since the sensitivity increases but no information regarding the effect on the noise is provided [23].

The results in [14] were obtained with a four-wire sensor head after 10 minutes of Joule annealing of the amorphous wire. The purpose of annealing the magnetic wire is to increase its circumferential anisotropy. When a 370 mA DC current is injected into the wire, the temperature is increased by Joule heating (rising to an estimated value of 170 ${}^{\circ }C$), and, simultaneously, the current generates a circumferential magnetic field. At the end of the annealing process, the magnetization will lay more favourably in the circumferential direction. Details of the annealing process can be found in [24]. As the annealing time is increased, the circumferential anisotropy increases and the noise in the 1/f region decreases. This is due to the fact that the 1/f noise of an orthogonal fluxgate in fundamental mode is mainly due to the Barkhausen caused by rapid domain wall movements in the core. Large circumferential anisotropy reduces such a domain wall movement and therefore Barkhausen noise.

In order to decrease the noise further, we tried to anneal the magnetic wires for a longer time, up to 60 min. Since crystalization temperature is never reached during annealing, we expected larger circumferential anisotropy and therefore lower noise. However, to much of our surprise, on the contrary, the 1/f noise increased, and eventually it became even bigger than the noise of sensors based on as-cast wires, making the annealing process counter-productive. Therefore, we decided to investigate the origin of this phenomenon and understand why the noise of the orthogonal fluxgate increases for long-time annealing and search for a method to solve this problem, which is presented in this paper.

## 2. Dependence of the 1/f Noise on the Annealing Time

As a first test, we built sensors based on a single AC20 (Unitika) wire with composition ${\left({\mathrm{Co}}_{0.94}{\mathrm{Fe}}_{0.06}\right)}_{72.5}{\mathrm{Si}}_{12.5}B_{15}$. As we used only one ferromagnetic wire in the fluxgate, the noise presented in this paper is about twice the noise of the previously mentioned sensors based on a four wire core, because the noise drops as the square root of the number wires is employed in the core. We annealed the wire in a four layer shielding using 370 mA dc current, flipping its polarity every second. We then used the wire as the core of orthogonal fluxgates with a 500 turns pick-up coils (5 mm diameter) wound around it. The wire was 8 cm length and the pick up coil was 6 cm length. In this way, we excluded the terminations of the wire from the flux picked up by the coil. During annealing, there could be a temperature gradient at the ends of the wire due to larger thermal mass of the soldering metal used to connect the wire to the annealing circuit. The lower temperature at the terminations of the wire could lead to a lower annealing-induced anisotropy at the ends of the wire, but since we exclude them from the pick-up coil, this does not pose a problem. The circuits for the core excitation, signal conditioning, and demodulation of the first harmonic from the pick-up coil voltage are described in detail in [14].

**Figure 1:**
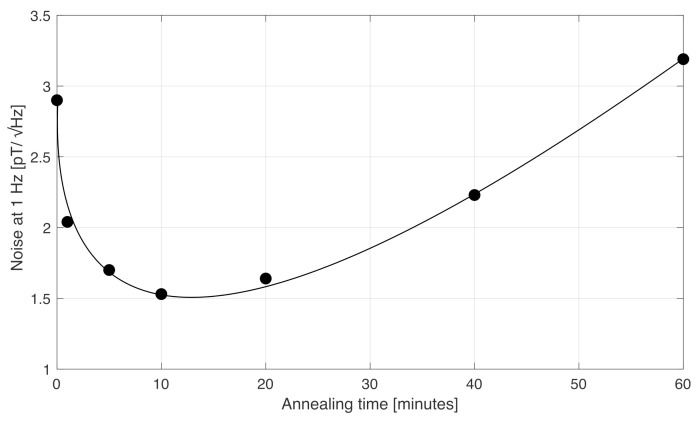
Dependence of the noise at 1 Hz of an orthogonal fluxgate in fundamental mode based on a single amorphous wire with ${\left({\mathrm{Co}}_{0.94}{\mathrm{Fe}}_{0.06}\right)}_{72.5}{\mathrm{Si}}_{12.5}B_{15}$ composition vs. annealing time.

Figure 1 shows the dependence of the noise level at 1 Hz on the annealing time. The noise was measured by inserting the sensor in a four-layer magnetic shielding. The excitation current was 40 mA peak at 30 kHz with a 50 mA dc bias. The output of the magnetometer was acquired by an ADS1299 digitizer. We should point out that we measured the noise on the very same wire. Values for different annealing times were obtained by progressively adding extra annealing time to the same wire already annealed. This was done because the noise can slightly vary from wire to wire, even if the portions of wires are taken from the same batch. Thus, a fair comparison can be obtained by only considering the same wire annealed for different times.

We can see that the sensor had a $2.8\;\;\mathrm{pT}/\sqrt{\mathrm{Hz}}$ noise at 1 Hz when based on the as-cast wire. The noise then decreased, reaching about $1.5\;\;\mathrm{pT}/\sqrt{\mathrm{Hz}}$ for 10 min annealing. However, when we further annealed the same wire for a longer time, the noise increased again, reaching $3.2\;\;\mathrm{pT}/\sqrt{\mathrm{Hz}}$ for 60 min annealing. This behaviour was repeatedly observed on different wires: for some wires, the minimum noise was obtained for 20 min annealing instead of 10 min, but generally the noise always rose for long-time annealing and, after 60 min annealing, always exceeded the noise of the sensor based on an as-cast wire.

**Figure 2:**
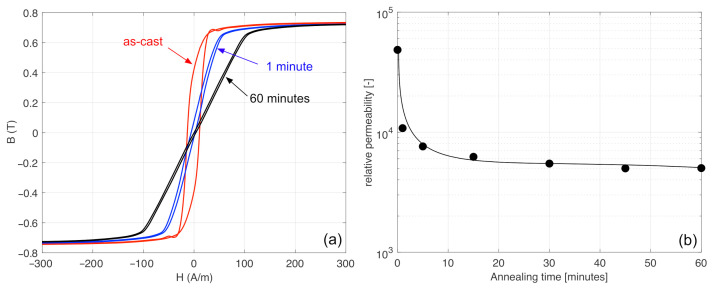
Axial B-H loop of Unitika AC20 wires in its as-cast form and for 1 min and 60 min annealing (**a**) and the corresponding axial relative permeability (**b**).

In order to understand the reason underlying this phenomenon, we first measured the longitudinal B-H loop of the magnetic wire for different annealing times. The B-H loop was measured using a conventional induction method. The wires were places in a 15 cm-long solenoid, which created an axial field on the wire, and the variation of the magnetic flux was obtained using a 3 cm-long pick-up coil wound around the the centre of the wire. The voltage induced in the pick-up coil was digitized and integrated numerically. The component of the flux in the air was subtracted numerically and the magnetic flux density was calculated after measuring the diameter of the microwire with a micrometer. As the anisotropy moves towards the circumferential direction, the axial B-H loop is supposed to show a gradual decrease of the permeability, and that is exactly what we observed. In Figure 2 we can see how the axial B-H loop was modified by annealing for 1 min and 60 min, compared to the B-H loop of the wire in its as-cast form. As we can see, the permeability after 60 min annealing was still lower than the permeability for 1 min annealing. In fact, we can see that the axial permeability monotonically decreased by increasing the annealing time. This indicates that the circumferential anisotropy always increases (even if slowly) as the annealing time increases. Thus, the annealing-induced anisotropy behaved as expected.

**Figure 3:**
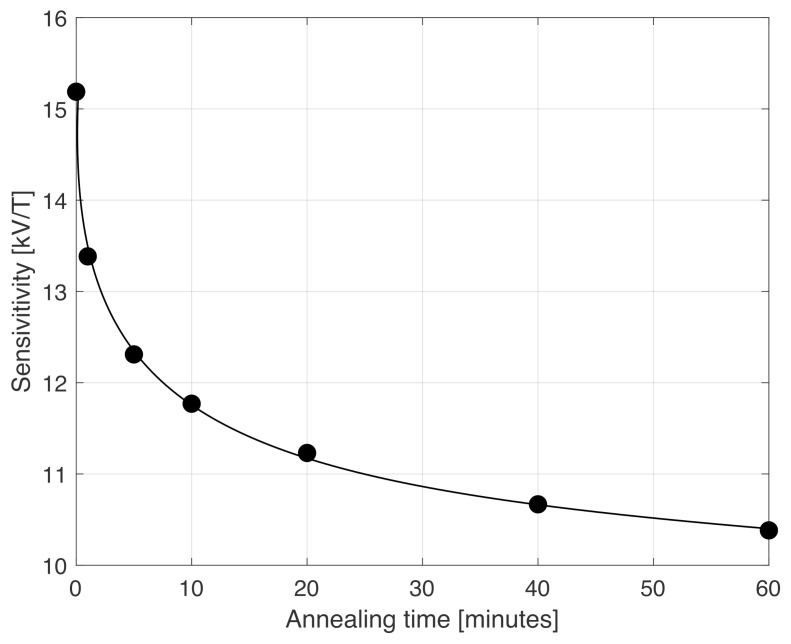
Sensitivity of the magnetometer before amplification for an orthogonal fluxgate in fundamental mode based on a single wire vs. annealing time.

Another possible reason for the increased noise after annealing the wires for a long time could be the change in sensitivity of the sensor. When the circumferential anisotropy of the wire increased, the sensitivity of the orthogonal fluxgates in fundamental mode decreased. This is simply due to larger circumferential anisotropy; the projection of the magnetization in the axial direction of the wire (i.e., the sensing direction) was lower, leading to a smaller voltage induced in the pick-up coil wound around the wire [25]. A small sensitivity could potentially lead to a situation in which the predominant noise of the magnetometer is not the noise of the sensor itself but rather the noise of the electronic (e.g., input amplifiers). Figure 3 shows the dependence of the sensitivity of the orthogonal fluxgate on the annealing time, as established in Helmholtz coils. While a sensor based on as-cast wire has a sensitivity above 15kV/T (before any amplification), the sensitivity monotonically drops down to a level just above 10kV/T after 60 min annealing. The whole electronic of the magnetometer [14] has an input-referred noise of $7\;\mathrm{nV}/\sqrt{\mathrm{Hz}}$ at 1 Hz at the working frequency: in the worst case scenario (10kV/T), this corresponds to $0.7\;\;\mathrm{pT}/\sqrt{\mathrm{Hz}}$ after demodulation. The noise of the electronics therefore is much lower than the total noise of the magnetometer ($3.2\;\;\mathrm{pT}/\sqrt{\mathrm{Hz}}$ for 60 min annealing). Here, we should point out that the noise of the electronic was uncorrelated to the sensor noise caused by Barkhausen noise in the magnetic core. Thus, these contributions have to be summed quadratically; this means that the noise of the magnetometer, once subtracting the noise of the electronics, was $\sqrt{3.2^{2}-0.7^{2}}=3.12\;\;\mathrm{pT}/\sqrt{\mathrm{Hz}}$, which was almost the same as the total noise $3.2\;\;\mathrm{pT}/\sqrt{\mathrm{Hz}}$. This means we were really observing sensor noise and not electronic noise.

This means that the reason for increased noise after long annealing is not the drop of sensitivity and it must originate somewhere else.

## 3. Influence of Annealing on the Magnetostriction

In a previous publication [26], we showed how the noise of an orthogonal fluxgate changes when we change the composition of the as-cast wire, modifying the quantity of cobalt and iron. We explained that the noise was minimum when the correct amount of cobalt and iron (94% of cobalt and 6% of iron of the total amount of cobalt plus iron) returned the minimum magnetostriction. As the amount of iron decreased below 6%, the magnetostriction became negative, whereas when the iron exceeded 6%, the magnetostriction turned positive. In both cases, the noise of the fluxgate increased, regardless of the sign of magnetostriction, because the minimum noise for a fluxgate was achieved when the absolute value of the magnetostriction was the lowest. This is due to the fact that any value of magnetostriction, either positive or negative, links the mechanical stress on the core to the magnetization, producing a change of the voltage induced in the pick-up coil. A similar behaviour was experimentally observed with second harmonic parallel fluxgates [27,28,29] and is the reason why the composition of amorphous tapes was fine tuned when stress annealing was used to reduce the Barkhausen noise.

However, the composition of the alloy is not the only factor determining the magnetostriction. In [30], Vazquez at al. show how current annealing for a long time can change the magnetostriction of a similar alloy. Specifically, they used ${\left({\mathrm{Co}}_{0.95}{\mathrm{Fe}}_{0.05}\right)}_{75}{\mathrm{Si}}_{10}B_{15}$ composition, which is a similar composition to ours, (${\mathrm{Co}}_{0.94}{\mathrm{Fe}}_{0.06}{)}_{72.5}{\mathrm{Si}}_{12.5}B_{15}$). In that paper, the authors show how long-time annealing affects not only the anisotropy but also the magnetostriction. The results were obtained using ribbons instead of wires, as in our case, but while this can have a significant difference on the anisotropy (because of different current distribution in the cross section of the ribbon compared to a wire), the effect of prolonged exposure to high temperature should not be different in wires compared to ribbons. In that paper, they show how the magnetostriction increased for different currents used for Joule annealing and for different annealing times, reaching an increase in magnetostriction of one order of magnitude when annealing time increased from 10 to 90 min, depending on the value of the current.

We believe this could have been the cause of the rising noise of our sensor when we annealed the wire for times longer than 30 min. Therefore, we measured the magnetostriction of the Unitika AC20 wires annealed with 370 mA for different durations. The magnetostriction measurements were carried out using the modified small-angle magnetization rotation (SAMR) method [31]. The wire was fed by an AC current of 20 mA–30 kHz, which was the excitation frequency of the sensor. The wire was inserted in a glass capillary (3 mm diameter), and a 200 turn coil was wound around it. A lock-in amplifier then demodulated the voltage induced in the pick-up coil to extract its second harmonic, $V_{2f}$. Simultaneously, a DC magnetic field $H_{Z}$ was applied in the axial direction using a 195 mm long coil. The field $H_{Z}$ was set to be large enough to saturate the magnetic wire in the axial direction. The amplitude of this field was in the order of hundreds of A/m, and it differed from measurement to measurement, since the annealing changed the anisotropy and therefore also the amplitude of the field necessary to saturate the wire in the axial direction. In all cases and for each experiment, we checked that the wire was axially saturated, verifying the linearity of the function $1/\sqrt{V_{2f}}$ vs. $H_{Z}$ as an indicator of saturation of the wire, as suggested in [31]. The amorphous wire was placed vertically inside the coil, generating $H_{Z}$, and it was connected to a support on its upper end while we applied weights with a mass in the order of tens of grams at its lower end in order to generate a mechanical tension to the wire. The strain applied to the wire spanned from 0 to 150 MPa. The measurement procedure was as follows: first, we started with no weights applied to the wire, $H_{Z}$ set to a value which saturated the wire, and we recorded the second harmonic $V_{2f}$ induced in the pick-up coil. Then, we increased the strain by adding weights in steps of Δσ and we changed $H_{Z}$ by the quantity $\Delta H_{Z}$ until we obtained the original value of $V_{2f}$ obtained with no weights applied. This means that the field $\Delta H_{Z}$ corresponded to the stress-induced anisotropy field. In turn, we can calculate the saturation magnetostriction coefficient $\lambda_{S}$ as:

(1) $$\lambda_{S}=\frac{\Delta H_{Z}\cdot M_{S}}{\Delta \sigma \cdot 3}$$

where $M_{S}$ is the saturation magnetization of the wire, as measured by a conventional induction method. 

**Figure 4:**
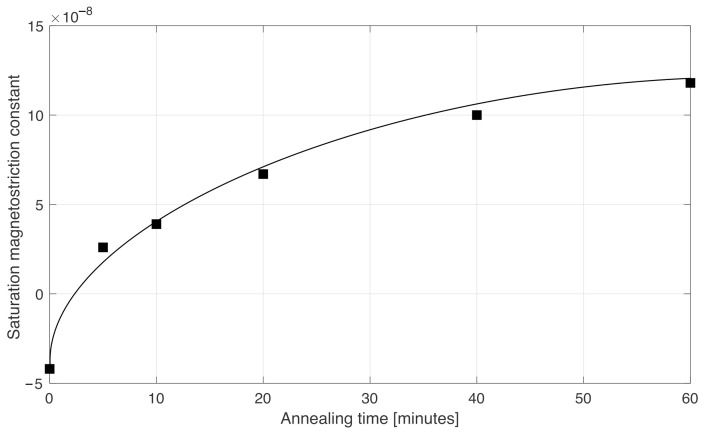
Saturation magnetostriction constant $\lambda_{S}$ of an Unitika wire with ${\left({\mathrm{Co}}_{0.94}{\mathrm{Fe}}_{0.06}\right)}_{72.5}{\mathrm{Si}}_{12.5}B_{15}$ composition vs. annealing time.

Figure 4 shows the dependence of $\lambda_{S}$ on the annealing time. As we can see, the as-cast wire had a slightly negative magnetostriction, about $\lambda_{S}=-4\times 10^{-8}$. As we annealled the wire, this value increased monotonically by more than one order of magnitude, until reaching $\lambda_{S}=+1.2\times 10^{-7}$ after 60 min. Quite interestingly, we observe that after 10 min, annealing the magnetostriction has the same absolute value as the as-cast wire, but with opposite sign ( $+4\times 10^{-8}$ instead of $-4\times 10^{-8}$), the noise of the sensor was lower because of the larger circumferential anisotropy. The problem arose when we annealed for a longer time because the circumferential anisotropy did not increase so rapidly any more, while the magnetostriction still rose. This is a first indication that the magnetostriction could be the cause of the rise of noise observed when we anneal the wire for more than 30 min. To fully confirm this theory, we did the following experiments.

**Figure 5:**
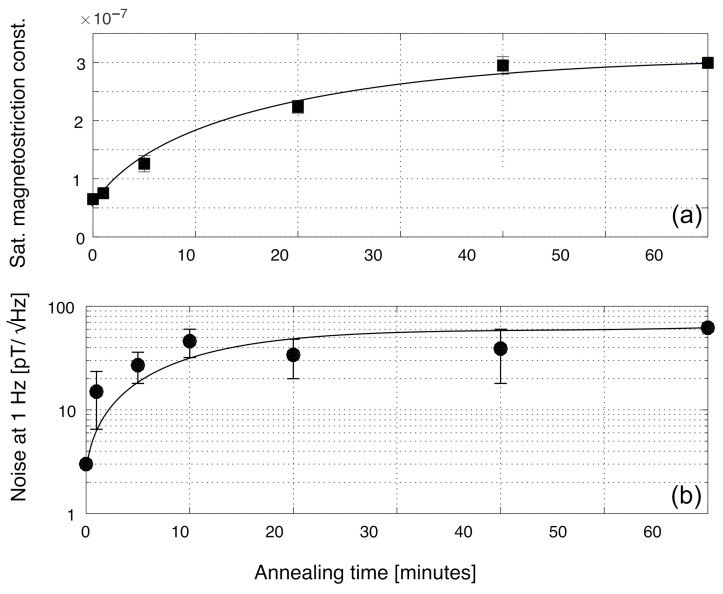
Saturation magnetostriction constant $\lambda_{S}$ (**a**) and noise (**b**) at 1 Hz of a wire with a nominal composition of ${\left({\mathrm{Co}}_{0.94}{\mathrm{Fe}}_{0.06}\right)}_{72.5}{\mathrm{Si}}_{12.5}B_{15}$ produced by the National Institute of Research and Development of Technical Physics of Iasi vs. annealing time, together with fitting errors.

First, we decided to repeat the same experiment using a wire from a different manufacturer, but with similar composition. The wire was produced by the National Institute of Research and Development for Technical Physics in Iasi, Romania. This wire should have the same nominal composition of the Unitika AC20 wires, that is ${\left({\mathrm{Co}}_{0.94}{\mathrm{Fe}}_{0.06}\right)}_{72.5}{\mathrm{Si}}_{12.5}B_{15}$. From the magnetostriction measurements, we found out that the magnetostriction was indeed slightly positive, starting at $\lambda_{S}=+6\times 10^{-8}$ for as-cast wire. In Figure 5a, we see that the magnetostriction monotonically increased as in the previous experiment: since it was already positive when the wire was in its as-cast form, by annealing the wire, the magnetostriction just grew, making the noise of the sensor worse. This was indeed what we observed in Figure 5b, where the noise at 1 Hz always increased with increase to the annealing time.

There were other sources of noise, for instance, thermal, vibrations, and residual Barkhausen noise, but they should not increase with increased annealing time. The only other possibility would be a slowly developed magnetic offset of the wire during the annealing (and its instability manifested as noise); however, with the annealing current being flipped, that should not be the case. It is possible that the noise increased because there was never a moment when the wire was annealed while having low magnetostriction, since the magnetostriction never crossed the zero. This means that any residual mechanical stress on the wire could additionally increase the noise.

## 4. Modified Composition

If the main cause of the increased noise for long-time annealing was really the increase of magnetostriction, we should be able to prove it by reducing the noise using wires with a lower magnetostriction in the annealed state. That could be achieved by lowering the content of iron in the alloy, with respect to the total amount of iron plus cobalt. The resulting as-cast magnetostriction is expected to be significantly negative [26], but as we perform annealing, the magnetostriction should gradually increase and eventually approach zero. This means that for long-time annealing we could combine both advantages: a larger circular anisotropy which reduces the Barkhausen noise on one hand and a vanishing magnetostriction on the other. As a result, we should not observe increasing noise for long-time annealing.

In order to test this hypothesis, we manufactured an amorphous wire at the Institute of Material Science of Madrid (CSIC), with a composition of ${\left({\mathrm{Co}}_{0.942}{\mathrm{Fe}}_{0.058}\right)}_{72.5}{\mathrm{Si}}_{12.5}B_{15}$. The diameter of the wire was about 120μm, similar to the diameter of the Unitika AC20 wires. We also measured its coercivity using a vibrating sample magnetometer, and it was only 63 A/m, indicating that the wire was completely amorphous.

**Figure 6:**
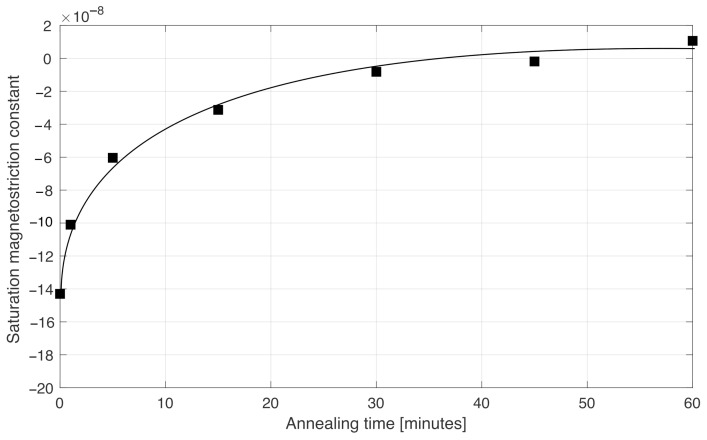
Saturation magnetostriction constant $\lambda_{S}$ (top) of a wire with a nominal composition of ${\left({\mathrm{Co}}_{0.942}{\mathrm{Fe}}_{0.058}\right)}_{72.5}{\mathrm{Si}}_{12.5}B_{15}$, produced at the Institute of Material Sciences of Madrid, vs. annealing time.

In this case, the content of iron, with respect to the total iron plus cobalt, was slightly lower than 6%, specifically, 5.8%. Thus, the magnetostriction was expected to be negative. This is, in fact, what we see in Figure 6: the magnetostriction of the as-cast wires reached $\lambda_{S}=-1.4\times 10^{-7}$. As we annealed the wire, the magnetostriction gradually increased and approached zero for an annealing time of about 45 min.

**Figure 7:**
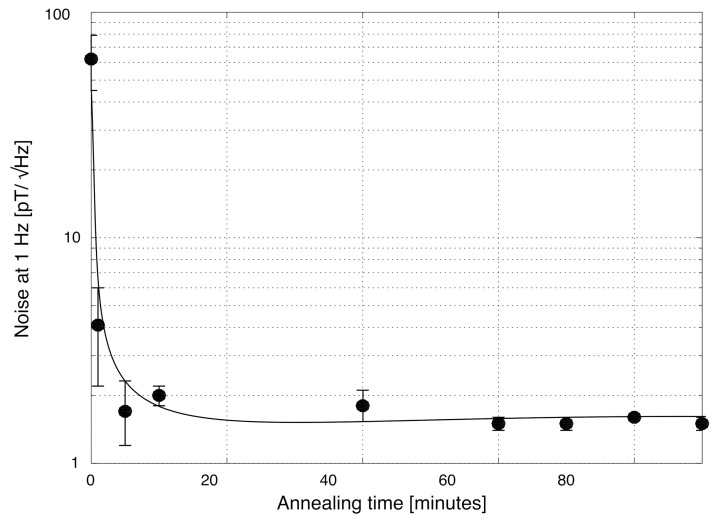
Noise at 1 Hz of a fluxgate magnetometer based on a wire with a nominal composition of ${\left({\mathrm{Co}}_{0.942}{\mathrm{Fe}}_{0.058}\right)}_{72.5}{\mathrm{Si}}_{12.5}B_{15}$, produced at the Institute of Material Sciences of Madrid vs. annealing time, together with fitting errors.

Following the results of the magnetostriction measurements, we expected the noise to simply decrease as we increased the annealing time, which is in fact what we observed. In Figure 7, we can see that, initially, the noise was very high for the as-cast wire, reaching $62\;\;\mathrm{pT}/\sqrt{\mathrm{Hz}}$ at 1 Hz. This was expected, because the absolute value of the magnetostriction was large. However, as we annealed the wire, the noise decreased, reaching $1.5\;\;\mathrm{pT}/\sqrt{\mathrm{Hz}}$ at 1 Hz, and, most importantly, it did not increase back for long-time annealing.

**Figure 8:**
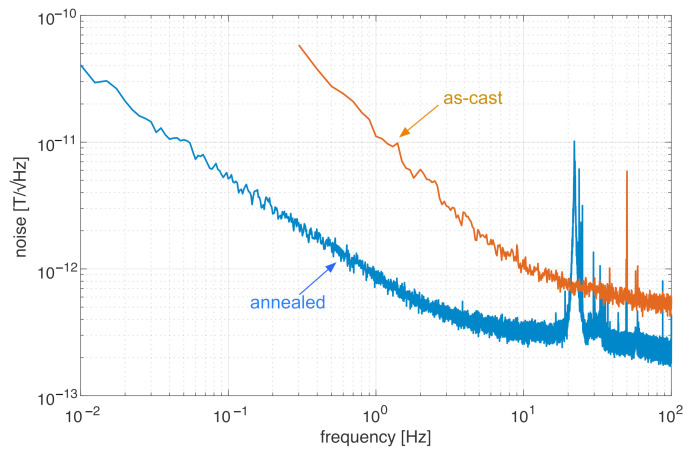
Noise spectra of a fluxgate magnetometer based on a wire with a nominal composition of ${\left({\mathrm{Co}}_{0.942}{\mathrm{Fe}}_{0.058}\right)}_{72.5}{\mathrm{Si}}_{12.5}B_{15}$ produced by the the National Institute of Research and Development of Technical Physics of Iasi, as-cast and after 5 min annealing.

In order to have an additional confirmation, we tested similar wires with the ${\left({\mathrm{Co}}_{0.942}{\mathrm{Fe}}_{0.058}\right)}_{72.5}{\mathrm{Si}}_{12.5}B_{15}$ modified composition produced by the National Institute of Research and Development for Technical Physics in Iasi, and we achieved similar results. We manufactured eight fluxgates using different sections of this wire, and for all of them, we obtained a large noise in their as-cast form (accounting for an average noise of $18\;\;\mathrm{pT}/\sqrt{\mathrm{Hz}}$ at 1 Hz, with a large variance from sample to sample), while the annealing dramatically decreased the noise. Quite interestingly, the noise obtained with these wires dropped down, after annealing for 5 min, to even less than $1\;\;\mathrm{pT}/\sqrt{\mathrm{Hz}}$ at 1 Hz (Figure 8). Such a low noise level has never been achieved for sensors based on a single Unitika AC20 wire (in order to achieve this noise using Unitika cores, a four-wire sensor head was required, with a disadvantage of larger power consumption and complexity). Both experiments indicate that it is really beneficial to start from a composition with a lower amount of iron and, therefore, a larger negative magnetostriction, as then the magnetostriction will be brought close to zero with annealing of the wire.

## 5. Additional Advantage of Long-Time Annealing

**Figure 9:**
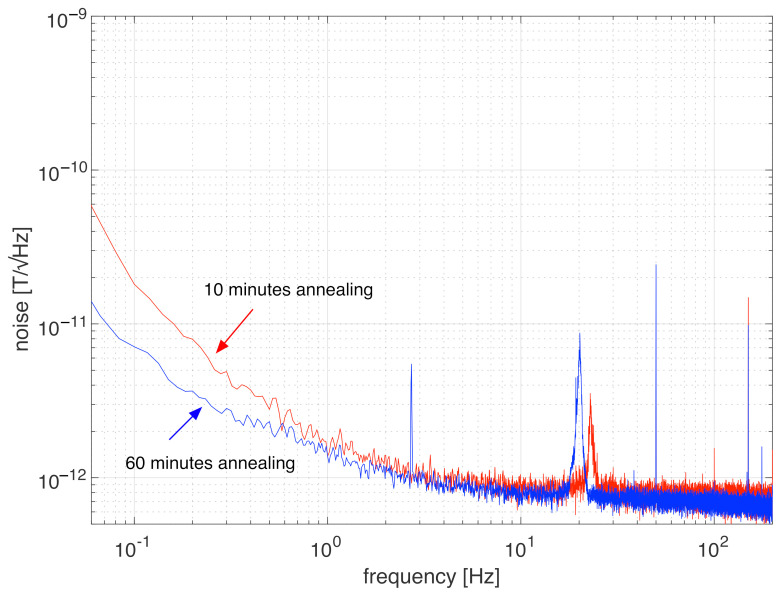
Noise spectra of two orthogonal fluxgates based on a single wire with ${\left({\mathrm{Co}}_{0.942}{\mathrm{Fe}}_{0.058}\right)}_{72.5}{\mathrm{Si}}_{12.5}B_{15}$ composition annealed for 10 min and 60 min.

In the previous sections, we presented the noise of different sensors as an amplitude spectral density at 1 Hz, which is a common standard used to evaluate the 1/*f* noise of magnetic sensors [32]. We can see that, by modifying the composition of the wires to ${\left({\mathrm{Co}}_{0.942}{\mathrm{Fe}}_{0.058}\right)}_{72.5}{\mathrm{Si}}_{12.5}B_{15}$, we obtained fluxgates whose noise no longer increased for long-time annealing. The noise obtained at 1 Hz, however, while not increasing, did not significantly decrease even when we performed long-time annealing (Figure 7), and this could lead to satisfactory results even with short-time annealing. However, the actual advantage of long-time annealing can be observed when considering the noise in the low frequency range (at 100 mHz and lower). In Figure 9, we can see the noise spectra of two sensors based on a wire with ${\left({\mathrm{Co}}_{0.942}{\mathrm{Fe}}_{0.058}\right)}_{72.5}{\mathrm{Si}}_{12.5}B_{15}$ composition produced at the Institute of Material Science of Madrid for annealing times of 10 min and 60 min. We can see that the second sensor, with 60 min annealing, had lower noise at 1 Hz (the difference was not so large because 1 Hz is very close to the corner frequency, which marks the beginning of the noise floor). However, when we observe the noise at frequencies lower than 1 Hz, we realise that it is significantly lower for the sensor with modified composition and long-term annealing (about 2.5 times lower at 100 mHz). Thus, we can conclude, that if we are interested in further reducing the noise of fundamental mode orthogonal fluxgates in the low frequency range, the modified composition with long-time annealing is the most favourable solution. The feasibility of long annealing for reduced noise at even lower frequencies (about 1 mHz) is an open question because of the temperature dependence of the fluxgate output, and more investigation is necessary in this field.

## 6. Conclusions

In this paper, we showed that the cause for low-frequency noise increase in fundamental mode orthogonal fluxgate when the core is annealed for a long time is the increase of magnetostriction. This problem can be solved by using magnetic wires as the core of the fluxgate with modified composition, namely ${\left({\mathrm{Co}}_{0.942}{\mathrm{Fe}}_{0.058}\right)}_{72.5}{\mathrm{Si}}_{12.5}B_{15}$. While this composition has a larger negative magnetostriction in its as-cast condition, the magnetostriction will eventually approach zero magnetostriction after 1 h of annealing. As a result, the magnetic wire will both have large circumferential anisotropy and zero magnetostriction. Thus, we suggest the use of this new composition for microwires to be annealed and used as the core of orthogonal fluxgates. A further development of this project is to understand the role of the evolving magnetostriction during the annealing process.

## Data Availability

The data presented in this article is available via the corresponding author.
